# Differential Effects of Cytopathic Hypoxia on Human Retinal Endothelial Cellular Behavior: Implication for Ischemic Retinopathies

**DOI:** 10.3390/ijms23084274

**Published:** 2022-04-12

**Authors:** Shaimaa El-tanani, Thangal Yumnamcha, Lalit Pukhrambam Singh, Ahmed S. Ibrahim

**Affiliations:** 1 Department of Ophthalmology, Visual, and Anatomical Sciences, School of Medicine, Wayne State University, Detroit, MI 48201, USA; hb3938@wayne.edu (S.E.-t.); gl5948@wayne.edu (T.Y.); plsingh@med.wayne.edu (L.P.S.); 2 Department of Clinical Pathology, Faculty of Medicine, Mansoura University, Mansoura 35516, Egypt; 3 Department of Biochemistry, Faculty of Pharmacy, Mansoura University, Mansoura 35516, Egypt; 4 Department of Pharmacology, School of Medicine, Wayne State University, Detroit, MI 48201, USA

**Keywords:** ischemic retinopathies, cytopathic hypoxia, human retinal endothelial cells (HRECs), CoCl_2_, seahorse technology, capacitance, impedance, ECIS modeling, R_b_ resistance, α resistance, barrier integrity

## Abstract

Loss of barrier integrity of retinal endothelial cells (RECs) is an early feature of ischemic retinopathies (IRs), but the triggering mechanisms remain incompletely understood. Previous studies have reported mitochondrial dysfunction in several forms of IRs, which creates a cytopathic hypoxic environment where cells cannot use oxygen for energy production. Nonetheless, the contribution of cytopathic hypoxia to the REC barrier failure has not been fully explored. In this study, we dissect in-depth the role of cytopathic hypoxia in impairing the barrier function of REC. We employed the electric cell-substrate impedance sensing (ECIS) technology to monitor in real-time the impedance (Z) and hence the barrier functionality of human RECs (HRECs) under cytopathic hypoxia-inducing agent, Cobalt(II) chloride (CoCl_2_). Furthermore, data were deconvoluted to test the effect of cytopathic hypoxia on the three key components of barrier integrity; R_b_ (paracellular resistance between HRECs), α (basolateral adhesion between HRECs and the extracellular matrix), and C_m_ (HREC membrane capacitance). Our results showed that CoCl_2_ decreased the Z of HRECs dose-dependently. Specifically, the R_b_ parameter of the HREC barrier was the parameter that declined first and most significantly by the cytopathic hypoxia-inducing agent and in a dose-dependent manner. When R_b_ began to fall to its minimum, other parameters of the HREC barrier, including α and C_m_, were unaffected. Interestingly, the compromised effect of cytopathic hypoxia on R_b_ was associated with mitochondrial dysfunction but not with cytotoxicity. In conclusion, our results demonstrate distinguishable dielectric properties of HRECs under cytopathic hypoxia in which the paracellular junction between adjacent HRECs is the most vulnerable target. Such selective behavior could be utilized to screen agents or genes that maintain and strengthen the assembly of HRECs tight junction complex.

## 1. Introduction

The retina is a multi-layered structure that lines the back of the eyeball of most vertebrates. Because of the high metabolic activity of the retina, the retina has two independent vascular systems. These systems are the retinal vasculature networks, which supply the inner two-thirds of the retina, and the choroidal system, which supplies the outer one-third of the retina [1]. Retinal Endothelial Cells (RECs) form a simple layer of squamous cells lining the inner surface of retinal vasculature networks, where they form the inner blood-retinal barrier (iBRB) [1]. The integrity of the REC layer is critical for regulating substances that can pass through iBRB as well as for preventing harmful chemicals and plasma components from entering the retina [2]. When the integrity of the REC layer is compromised, the retinal functionality is disrupted, resulting in various retinal blinding diseases, including ischemic retinopathies (IRs) such as retinopathy of prematurity (ROP), diabetic retinopathy (DR), and neovascular age-related macular degeneration (AMD) [2].

IRs are visual diseases defined by ischemia in the first phase, followed by aberrant neovascularization in the second phase, leading to retinal detachment and blindness [3,4]. Despite the benefits of photocoagulation [5] and anti-VEGF therapies [6,7,8] in treating the neovascularization phase, there is still a need to find new targets that prevent the early stages of these ocular diseases [9,10,11]. In IRs, retinal ischemia is caused by damage to mature retinal vessels (as in many cases of diabetic retinopathy, retinal vein occlusion, or sickle cell retinopathy) or immature retinal vasculature (as in retinopathy of prematurity) [3]. However, the triggering mechanism of REC damage that causes retinal ischemia remains incompletely understood. Previous studies have reported mitochondrial dysfunction in several forms of IRs [12,13,14], suggesting that malfunctioned mitochondria damage REC by creating a cytopathic hypoxic environment, where cells are unable to use oxygen for energy production. Nonetheless, the contribution of cytopathic hypoxia to REC barrier failure has not been fully explored.

The hypoxia mimicking agent, cobalt chloride (CoCl_2_), is frequently utilized to study a variety of ischemic diseases, including ischemic brain injury [15] and retinal ischemia [16]. CoCl_2_ induces cytopathic hypoxia by its competitive inhibitory effect on multiple iron-dependent proteins linked with the mitochondrial electron transport chain (ETC) [17,18]. Furthermore, CoCl_2_ inhibits prolyl hydroxylases (PHDs) that regulate the stability of hypoxia-inducible factors (HIFs), master regulators of cellular hypoxic response [19]. Accordingly, the use of CoCl_2_ provides a suitable model to dissect the role of cytopathic hypoxia in compromising the REC barrier integrity.

**Figure 1:**
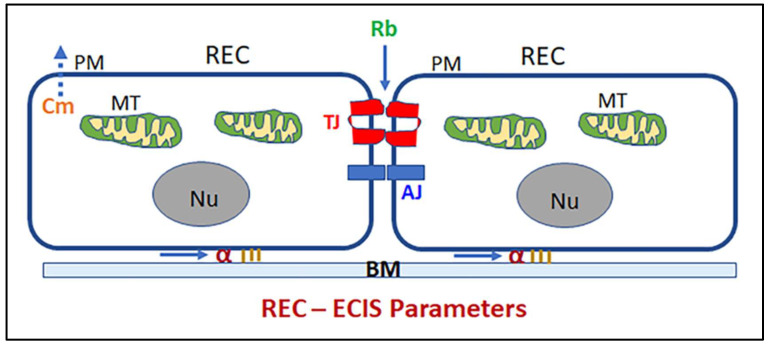
Diagram showing ECIS parameters in the retinal endothelial cell (REC). R_b_: paracellular resistance between RECs; α: basolateral resistance between REC and the extracellular matrix; C_m_: cell membrane capacitance. MT: mitochondria; PM: plasma membrane; Nu: nucleus; TJ: tight junction; AJ: adherens junction; BM: basement membrane.

Given the importance of preserving the REC of iBRB to the retinal functionality [20], the ability to dynamically evaluate how REC barrier function changes when subjected to cytopathic hypoxia would be a valuable tool. We have shown the usefulness of the Electric Cell-substrate Impedance Sensing (ECIS) system in real-time monitoring the effect of cytopathic hypoxia on the barrier integrity of retinal pigment epithelium an essential component of the outer BRB [21]. ECIS system is a biosensor multitasking system that continuously monitors changes in cells’ behavior and models many important parameters that describe cellular barrier integrity [22]. By using alternating current (AC) instead of direct current (DC), ECIS has the capability of dissecting the components of RECs’ total impedance (Z) into barrier resistance (R) and capacitance (C), which are frequently used to monitor barrier functionality and cellular spreading over the substrate, respectively. Another important feature of ECIS is the inclusion of multifrequency measurement along with the usage of AC [23]. This feature allows the mathematical modeling of the overall cellular measurements into three distinct parameters related to barrier integrity (Figure 1); R_b_ (paracellular resistance between RECs), α (basolateral resistance between RECs and the extracellular matrix), and C_m_ (REC membrane capacitance). As a result, the application of ECIS Technology presents a valuable platform to non-invasively analyze the barrier functionality of RECs under ischemic conditions.

The goals of the current study were to determine the effect of cytopathic hypoxia on the behavior of RECs and to evaluate the quality of endothelial paracellular junctions, endothelial cell membrane, as well as the adhesion between endothelial cells and extracellular matrix under the condition of cytopathic hypoxia.

## 2. Results 

### 2.1. Effect of Cytopathic Hypoxia on HREC Electrical Impedance

**Figure 2:**
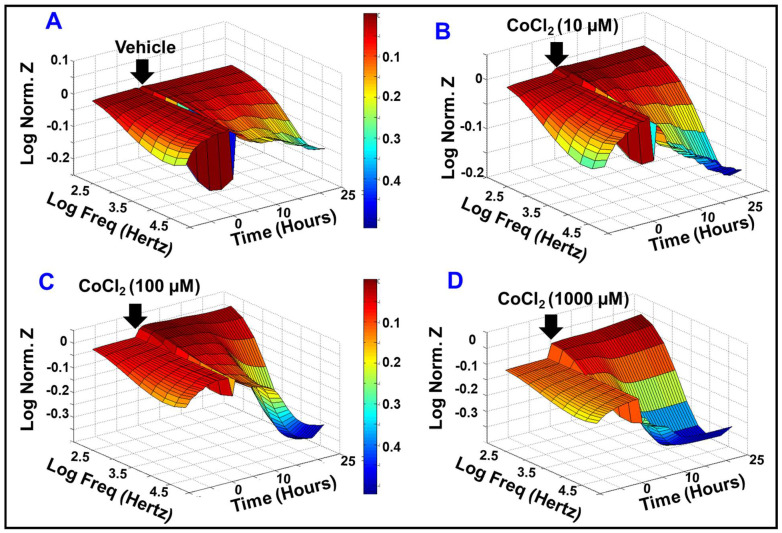
Three-dimensional plots of the log of normalized impedance (Z) across HRECs against time and the log of the alternating-current (AC) frequency applied to the ECIS electrode. The control vehicle or different CoCl_2_ treatment were added at the time (t) = 0, which was 44.8 h after culturing HRECs on the ECIS electrode. After treatment, Zt was tracked for 25 h, where Z0 at t = 0 was used to normalize all other Z measurements, such that each reading was calculated as the log of the ratio of Zt/Z0. The maximum value is when Zt = Z0 (hence the ratio becomes 1, and the log equals 0). Abbreviations: Z: impedance; Norm: normalized; Freq: frequency; Zt: the impedance at time t; Z0: the impedance at time 0.

Given that IRs are related to retinal endothelial dysfunction, the role of cytopathic hypoxia in impairing HREC barrier functionality was investigated in a real-time manner using ECIS^®^ instrumentation. In this experiment (Figure 2), various concentrations (0, 10, 100, and 1000 µM) of cytopathic hypoxia-inducing agent (CoCl_2_) were applied to HRECs after the impedance (Z) reached the plateau phase, where HRECs form stable and confluent monolayer with mature tight junctions. Then the barrier integrity of HRECs was evaluated based on total Z over 25-h across a frequency range of 250 to 64,000 Hz. As indicated in Figure 2A–D, CoCl_2_-treatment resulted in a dose-dependent reduction in overall Z of HRECs across all measured frequencies, implicating an important role of cytopathic hypoxia in impairing the barrier functionality of HRECs.

**Figure 3:**
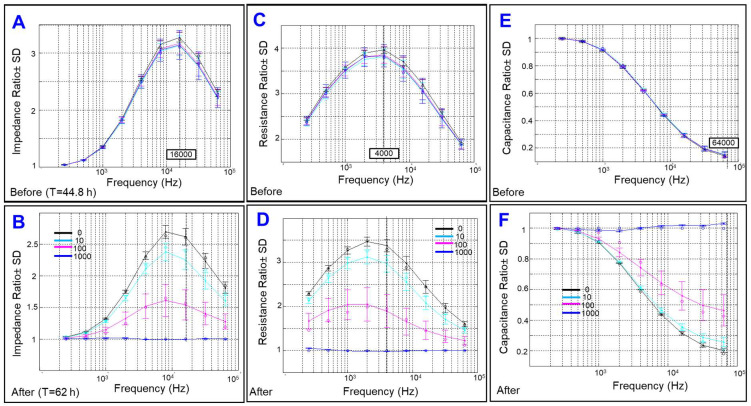
Impedance, resistance, and capacitance ratios between HRECs and the cell-free medium against different frequencies were measured at 44.8 h after placing HRECs on ECIS electrodes and before adding CoCl_2_ (**A**,**C**,**E**, respectively) or at 17 h post-CoCl_2_ treatment, ranging from 0 to 1000 µM (**B**,**D**,**F**, respectively) measured at 62 h after placing HRECs on ECIS electrodes. Local maximums for impedance, resistance, and capacitance were at 16,000, 4000, and 64,000 Hz, respectively.

Next, since Z of cells consists of cell barrier resistance (R) and cell membrane capacitance (C), we determined whether cytopathic hypoxia affects one or both components of Z. Generally when cells are subjected to alternating current (AC), both R and C are generated with Z as an endpoint. However, when cells are subjected to direct current (DC), the C vanishes, and R is equivalent to Z. Taking advantage of ECIS as an AC applying system with frequency-dependent amplitudes, simultaneous measurements of R and C were obtained at each specific frequency [22]. To determine the best fit frequency to be utilized in subsequent evaluations of cytopathic hypoxia effects on Z components, the frequency dependency spectra of Z components were first determined. Figure 3A,C,E illustrate the frequency dependence spectra of the Z, R, and C, respectively, for HRECs at the time (t) = 44.8 h after placing HRECs onto the ECIS array and just before starting any treatment. At this time, HRECs were confluent, and the Z spectrum exhibited a distinctive frequency of 16,000 Hz, where the maximum Z ratio was obtained between wells with HRECs versus those without cells (Figure 3A). In addition, this frequency gives the broadest range for comparing different post-CoCl_2_-treatment groups (Figure 3B).

Regarding the frequency dependence spectrum of the R (Figure 3C), it showed that at a frequency of 4000 Hz, a maximum R ratio between wells with HRECs versus those without cells was obtained, providing the broadest possible range for the comparison between post-CoCl_2_-treatment groups (Figure 3D). Therefore, this frequency was chosen for subsequent R analysis. Lastly, the frequency dependence spectrum of C (Figure 3F) showed a frequency of 64,000 Hz to give a broadest possible range to compare the C among different CoCl_2_-treatment groups and thereby was utilized in the subsequent C analysis.

### 2.2. Effect of Cytopathic Hypoxia on the Capacitance of HRECs

**Figure 4:**
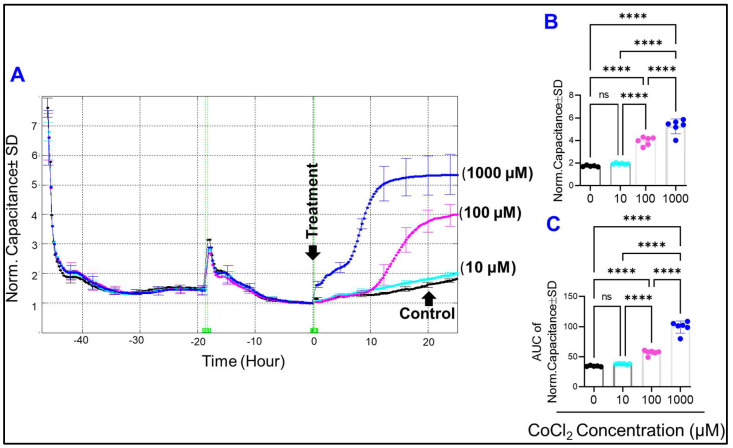
(**A**) Normalized capacitance of HREC groups vs. time at a frequency of 64,000 Hz. Different concentrations of CoCl_2_ were applied at t = 0. Experimental groups are control, 10 µM CoCl_2_, 100 µM CoCl_2_, and 1000 µM CoCl_2_. (**B**) Statistical analysis of each group’s normalized capacitance at the end of the experiment (t= 25 h). (**C**) Statistical analysis of each group’s area under the normalized capacitance curve between the interval t = 0 and t = 25 h. The ANOVA test followed by the Tukey post-hoc test was used for statistical comparison between the experimental groups. Abbreviations: Norm: normalized; AUC: area under the curve. **** *p* ≤ 0.0001; ns: no significance.

Given that the spreading of cells on substrates impacts their behavior, the ability of HRECs to spread over ECIS electrodes under cytopathic hypoxia was also evaluated. In this regard, the C of HRECs was measured because of the inverse relationship between the C and cells’ spreading [23]. To do so, the frequency at which the maximum spreading of HRECs could be detected was chosen to be the one that gives the lowest C, which corresponds to 64,000 Hz in Figure 3E. This inverse relationship was confirmed in Figure 4A, where the C of HRECs at 64,000 Hz exhibited a decreasing tendency until it reached a plateau after 10–15 h of placing HRECs on ECIS electrode, reflecting a fully spread stage of HRECs. Then the media was changed into serum-free media to prevent cell proliferation within the experimental period. After that, different concentrations of CoCl_2_ were applied. As shown in Figure 4A, the C of HRECs increased instantly after CoCl_2_ (1000 µM) treatment, whereas it took 8–12 h for the C of HRECs to rise in response to 100 µM of CoCl_2_. In contrast, CoCl_2_ at 10 µM had no significant effect on the C of HRECs compared to the controls. Notably, 100 µM and 1000 µM of CoCl_2_ resulted in significant increases in the C of HRECs both at the end (t = 25 h post-treatment, Figure 4B) and throughout the experiment (evaluated by the AUC, Figure 4C) in a dose-response manner. These data collectively demonstrate that only higher levels of cytopathic hypoxia affect how HRECs spread over the substrate.

### 2.3. Effect of Cytopathic Hypoxia on the Total Resistance of HRECs

**Figure 5:**
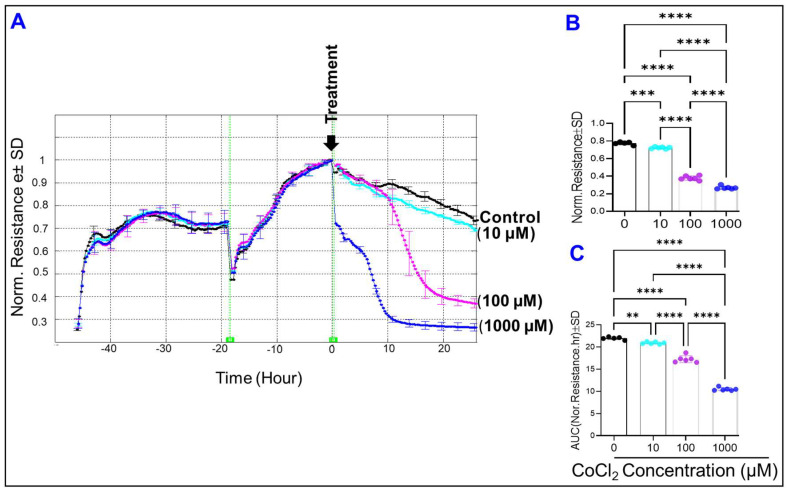
(**A**) Normalized resistance of HREC groups over time, using a frequency of 4000 Hz. Treatments were applied at t = 0, and resistance measurements were taken starting from placing HRECs onto the ECIS electrode until 25 h after treatment application. HREC experimental groups are control (0), 10 µM CoCl_2_, 100 µM CoCl_2_, and 1000 µM CoCl_2_. (**B**) Statistical analysis of each group’s normalized resistance at the end of the experiment, at t = 25. (**C**) Statistical analysis of each group’s area under the normalized resistance curve for the interval between t = 0 and t = 25. Statistical analysis was performed using the ANOVA test followed by the Tukey post hoc test. Abbreviations: Norm: normalized; AUC: area under the curve. ** *p* ≤ 0.01; *** *p* ≤ 0.001; **** *p* ≤ 0.0001.

To analyze the effect of cytopathic hypoxia on the barrier function of HRECs, the R parameter of the Z across the cell monolayer was measured at 4000 Hz, a frequency at which the R is maximum (Figure 3C)**.** As shown in Figure 5A, treating HRECs with CoCl_2_ resulted in declines of R over time in a dose-dependent fashion. The decrease in R began with a 1000 µM concentration of CoCl_2_ as it quickly reached a minimum level within 10 h of the treatment. The following change in the R was observed with 100 µM of CoCl_2_ treatment, evident by a gradual and continuous drop in the R curve until it reached its minimum at 25 h post-treatment. The 10 µM CoCl_2_ group was the last to have considerable losses in R compared to the control group. Interestingly, all concentrations of CoCl_2_ showed significant reductions in R compared to the control group at the end of the experiment (Figure 5B) as well as throughout the experiment (Figure 5C) in a dose-dependent fashion, where each treatment group experienced R losses at rates significantly different from each other and from the control group. These results indicate that cytopathic hypoxia, at all concentrations, compromises the barrier integrity of HRECs.

### 2.4. Effects of Cytopathic Hypoxia on Components of Transendothelial Resistance of HRECs

**Figure 6:**
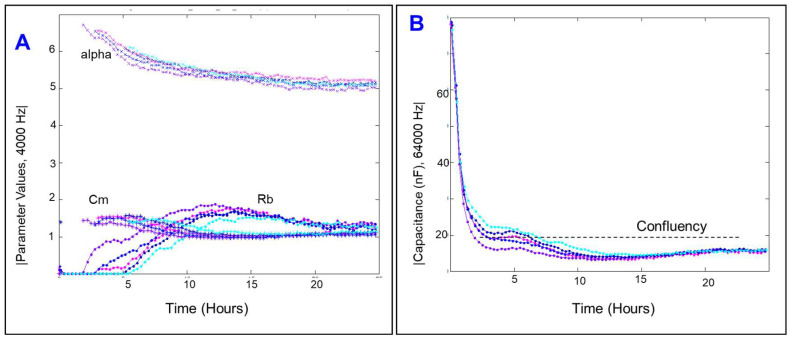
(**A**) Deconvolution of the total resistance across the HRECs into three separate components: α, the resistance between the cells and their basolateral substrate; R_b_, the resistance paracellularly; and C_m_, cell plasma membrane capacitance. The α, R_b_, and C_m_ of HRECs were measured at 4000 Hz during the first 25 h after culturing HRECs onto the electrodes. (**B**) The capacitance of HRECs vs. time at 64,000 Hz was measured for 25 h after the first culture of HRECs onto the electrodes; *n* = 5.

Because the transendothelial resistance comprises three components: R_b_, α, and C_m_, we sought to determine whether cytopathic hypoxia has global or differential effects on these components in HRECs. To achieve this goal, the obtained data at 4000 Hz were first deconvoluted into three separate curves representing these components using the mathematical model of Giaever and Keese [22]. As shown in Figure 6A, adhesion of HRECs to their substrate (represented by α) and stable C_m_ were achieved first at ~2 h, followed by spreading of HRECs out and monolayer formation by ~7 h, evident by reaching a plateau in the C curve of HRECs (Figure 6B). R_b_ values did not start to model until ~5 h after HRECs were placed on ECIS electrodes and peaked at ~10–12 h (Figure 6A). These data demonstrate that the establishment of α, C_m_, and monolayer confluency for this specific cell type is necessary to achieve durable R_b_ and a mature barrier function.

**Figure 7:**
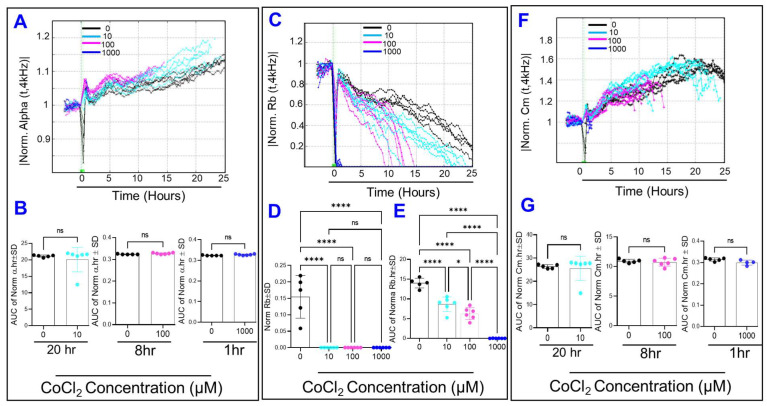
(**A**) Normalized α vs. time from the time of treatment to t = 25 h post-treatment. (**B**) Areas under the α curves for the intervals when the α value can be modeled in each group. CoCl_2_ treatment is associated with no α changes from control in each of the groups along the interval for each when their alpha values were able to be calculated (t = 0–20 h for the 10 µM, t = 0–8 h for the 100 µM, and t = 0–1 h for the 1000 µM). (**C**) Normalized R_b_ vs. time curves. (**D**) Bar chart of each group’s normalized resistance at t = 25 h. (**E**) Areas under the normalized R_b_ curves from t= 0–25 h. ANOVA test followed by Tukey post hoc test for all groups demonstrates how R_b_ responds to CoCl_2_ in a dose-dependent manner over the experimental duration, with an inverse relationship between CoCl_2_ concentration and R_b_ AUC. (**F**) Normalized C_m_ vs. time curves. The ECIS is unable to calculate a real value for C_m_ at time points when R_b_ is at or below zero. (**G**) Areas under the normalized C_m_ curves for the intervals when the C_m_ value can be modeled in each group. CoCl_2_ treatment is associated with no C_m_ changes from control in each group along the interval for each their C_m_ values can be calculated (t = 0–20 h for the 10 µM, t = 0–8 h for the 100 µM, and t = 0–1 h for the 1000 µM). Statistical analysis was performed using the ANOVA test followed by the Tukey post hoc test. Abbreviations: Norm: normalized; AUC: area under the curve; ns: no significance; *: *p* ≤ 0.05; ****: *p* ≤ 0.0001; *n* = 5–6/group.

After dissecting transendothelial resistance of HRECs into its three components, the effect of cytopathic hypoxia on each of these components was then evaluated (Figure 7). Firstly, α curves in Figure 7A for 1000, 100, and 10 µM of CoCl_2_ were terminated at (0–1 h), (10–15 h), and (20–25 h), respectively, which are time periods when the corresponding R_b_ value becomes zero (Figure 7C). ECIS can only model α values if the corresponding R_b_ values are positive (not zero). During these experimental periods and before α curves ended, none of the CoCl_2_ concentrations had a significant effect on α values (Figure 7B). Secondly, Figure 7C shows the effect of cytopathic hypoxia on normalized R_b_ over time. While all concentrations of CoCl_2_ completely diminished the contribution of R_b_ to cell resistance at the end of the experiment, there was no noticeable dose-response effect (Figure 7D). However, monitoring the impact of CoCl_2_ on R_b_ behavior throughout the experiment with the calculation of AUC, dose-dependent reductions in R_b_ were detected (Figure 7E). Thirdly, Figure 7F displays C_m_ over time after treatment with CoCl_2_, and again, we noticed that C_m_ curves for 1000, 100, and 10 µM of CoCl_2_ were ended abruptly at (0–1 h), (10–15 h), and (20–25 h), respectively. These terminations of C_m_ curves were due to the fact that ECIS cannot estimate actual values for C_m_ when the corresponding R_b_ values are zero. Considering the AUC of C_m_ across the aforementioned time intervals and before C_m_ curves ended, none of the CoCl_2_ concentrations significantly affected C_m_ values (Figure 7G)**.** Collectively, the results in Figure 7 indicate that cytopathic hypoxia mainly affects the integrity of the paracellular junctions between HRECs as the R_b_ was the only component of the three to respond in a dose-dependent manner.

### 2.5. CoCl_2_ Compromises HRECs’ Mitochondrial Bioenergetics without Causing Cytotoxicity

**Figure 8:**
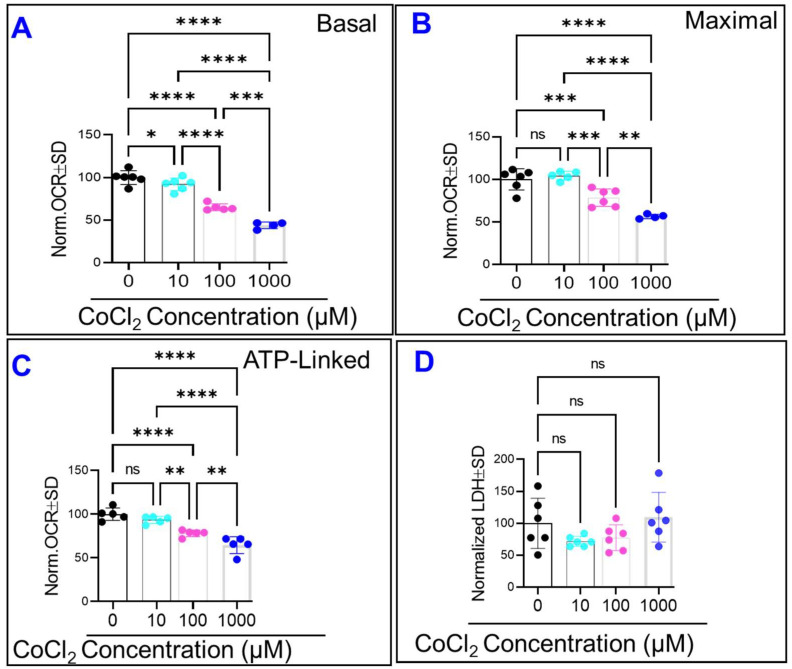
**Effects of cytopathic hypoxia on HREC mitochondrial bioenergetics.** HRECs were treated with varying concentrations of CoCl_2_ (0, 10, 100, and 1000 µM) for 24 h before evaluating oxygen consumption rate (OCR) with XFp Cell Mito Stress kit (**A**–**C**). Data are normalized means of OCR (pmol/minute) ± SD to control (30,000 cells/well). The optimal concentrations of oligomycin, FCCP, and antimycin/rotenone were titrated (1 μM/each) (data not shown). The 10 µM concentration of CoCl_2_ only impacted basal OCR and did not affect maximal or ATP-linked OCRs, whereas both the 100 µM and 1000 µM concentrations of CoCl_2_ significantly impaired basal, maximal, and ATP-linked OCRs of HRECs (**A**–**C**, respectively). None of the CoCl_2_ concentrations exerted a cytotoxic effect on HRECs, as the release of lactate dehydrogenase (LDH) in the supernatant was not changed at the end of the experiment (**D**). Ns: no significance; *: *p* ≤ 0.05; **: *p* ≤ 0.01; ***: *p* ≤ 0.001; ****: *p* ≤ 0.0001; *n* = 5–6/group.

To verify that the adverse effects of CoCl_2_ on HREC barrier function were associated with mitochondrial dysfunction, cellular oxygen consumption rates (OCRs) were analyzed using a Seahorse flux bioanalyzer (XFe96, Agilent, Santa Clara, CA, USA) and Mito-Stress test. First, as shown in Figure 8A, all tested concentrations of CoCl_2_ dose-dependently and significantly reduced HRECs’ basal OCRs, normalized by subtracting non-mitochondrial OCRs (corresponding to rotenone/antimycin-insensitive OCRs). Second, the ATP synthase inhibitor (oligomycin) was added to separate OCRs linked to ATP production by subtracting resultant OCRs from basal OCR. As shown in Figure 8B, only 100 and 1000 µM of CoCl_2_ lowered ATP-linked OCRs in HRECs significantly and in a dose-dependent manner compared to control. Third, the addition of protonophore uncoupler (FCCP) stimulated OCRs to their maximal activities in both control and CoCl_2_-treated HRECs but to a significantly lower extent in 100 and 1000 µM CoCl_2_-treated cells (Figure 8C). These results confirmed that all CoCl_2_ tested concentrations impaired mitochondrial respiratory function under basal conditions, creating a cytopathic hypoxia-like condition.

To further ensure that the observed effect of CoCl_2_ on barrier integrity was not a consequence of cell cytotoxicity, the LDH assay was performed at the end of the experiment. The LDH assay results in Figure 8D did not demonstrate any cytotoxicity at all tested concentrations of CoCl_2_ within the 24 h experimental period, where losses to total resistance have already occurred, particularly in 100 and 1000 µM CoCl_2_ groups (Figure 5). Altogether, these data indicate that the disruption of paracellular barrier integrity of HRECs is an earlier event that occurs in response to cytopathic hypoxia long before any noticeable effect on cell viability.

## 3. Discussion

The novel finding of the present study is that cytopathic hypoxia compromises the barrier integrity of HRECs in distinct ways, with the paracellular junction between adjacent HRECs being the most vulnerable target. This conclusion was based on real-time monitoring and dissecting of HREC barrier dysfunction induced by cytopathic hypoxia across the paracellular junction between HRECs (evaluated by R_b_), the HRECs-substrate basolateral adhesion (evaluated by α), and the barrier to flow through the cell membrane of HRECs (evaluated by C_m_). The following experimental results support this conclusion: (i) the R_b_ parameter of the HREC barrier was the parameter that declined first and most significantly by the cytopathic hypoxia-inducing agent and in a dose-dependent manner; (ii) during the period when R_b_ began to fall to it is minimum, other parameters of the HREC barrier, including α and C_m_, were unaffected, and (iii) intriguingly, the compromised effect of cytopathic hypoxia on R_b_ was associated with mitochondrial dysfunction but not with cytotoxicity. Using mathematical modeling of ECIS data, our study is the first to demonstrate these temporal correlations between the three components of the HREC barrier under the condition of cytopathic hypoxia. ECIS instrumentation is the only technology currently available that enables modeling each of these critical cellular barrier components. In terms of modeling endothelial barrier function in vitro, the R_b_ denotes paracellular permeability, governed by tight junction complexes. The component α reflects changes in cell basal adhesion, controlled by integrins, while C_m_ describes the changes in membrane composition as a function of capacitance.

Maintaining the endothelial barrier integrity is essential for the proper functioning of tissues and organs. Therefore, it is crucial to know which barrier parameters contribute to the endothelial barrier’s strength, and this will vary depending on endothelial cell type. For example, lymphatic endothelial cells are leaky because they are poor in cell-cell tight junction (TJ) structures, yet they demonstrated high barrier resistance on ECIS, which is derived mainly from α of cell-substrate adhesion [24,25]. On the other hand, brain endothelial cells are enriched in TJs and exhibit high R_b_ and α. In agreement, our study shows that HRECs have high barrier resistance on ECIS, which is derived from establishing robust α and C_m_ followed by a stable R_b_ [23]. Since the endothelial barrier of the retina is compromised in IRs, our study is the first to dissect which parameters of the HREC barrier are more sensitive to ischemic insults. The ECIS modeling software clearly indicates that the paracellular resistance (R_b_) is the only barrier parameter affected by the cytopathic hypoxia-inducing agent in a dose-dependent manner (Figure 7).

There are several mechanisms through which cytopathic hypoxia induces HREC dysfunctions that can be applied to changes in R_b_ seen in our model. One such mechanism refers to the upregulation of vascular endothelial growth factor (VEGF) by CoCl_2_ and activation of its cognate receptor (VEGFR2) [26,27]. Activation of *VEGFR2* phosphorylates a critical paracellular TJ protein that regulates R_b_, Zonula occludens (ZO)-1, and results in subsequent dissociation of ZO-1 from TJ complexes [28]. In agreement, VEGF receptor inhibitor (SU1498) improved blood-brain barrier maintenance under hypoxia [29]. Additionally, our previous study showed that CoCl_2_ reduces expression and disrupts the distribution of ZO-1 in retinal epithelial cells [25]. Therefore, it is plausible that phosphorylation of ZO-1 by the cytopathic hypoxia-driven VEGF causes remodeling of HRECs paracellular junctions and changes in R_b_ seen in our model.

The second mechanism that may contribute to the breakdown of the HREC paracellular barrier under cytopathic hypoxia is the generation of reactive oxygen species (ROS), particularly from mitochondrial dysfunction, that damages adherens junction proteins (AJPs). VE-cadherin and β-catenin are the major AJPs regulating paracellular permeability that are affected by ROS generation [30]. In addition, previous studies have shown that CoCl_2_ stabilizes HIF-1α expression [31,32,33], and increased HIF-1α levels are necessary to increase NADPH oxidase (Nox)2 expression and the subsequent ROS production [34], which promotes internalization of VE-cadherin along with β-catenin from the cell-cell contacts [35]. This internalization of *AJPs* results in adherens junction disassembly and thus may explain the compromise of R_b_ barrier integrity in HRECs subjected to cytopathic hypoxia.

An additional explanation for the breakdown of the HREC paracellular barrier under cytopathic hypoxia is the decreased ATP-linked OCR (Figure 8C), which describes damaged ETC and impaired ATP synthesis [36]. ATP depletion has been shown to cause tight junction disassembly by interfering with specific protein-protein interactions in the atypical protein kinase C (aPKC) signaling pathway. Under normal conditions, aPKC phosphorylates partitioning-defective (Par)3 at serine 827, which is vital in establishing and maintaining TJ assembly [37]. However, under ATP-depleting conditions, aPKC is unable to phosphorylate Par3 stabilizing the association of Par3 with aPKC, thereby preventing its release and localization to the tight junction [38]. Collectively, reduced ATP production with aberrant aPKC signaling may contribute to the loss of tight junctions and lower R_b_ values in HRECs subjected to cytopathic hypoxia.

## 4. Conclusions

Our results support that HRECs have discernible dielectric properties under cytopathic hypoxia, in which the disturbance of the paracellular barrier between adjacent HRECs is a prodromal index of impending endothelial dysfunction that occurs prior to any loss in cell viability and cell-substrate adhesion as well as prior to any changes in membrane composition. Future studies aimed at finding gene targets manipulating paracellular barriers of retinal endothelial cells subjected to ischemic insults could utilize the ECIS technology as a powerful tool *in screening* such genes. and, therefore, drug development.

## 5. Materials and Methods

### 5.1. ECIS Experiment and Modeling

The effects of cytopathic hypoxia on retinal endothelial cellular behaviors were assessed by monitoring the overall cellular impedance (Z) using (ECIS^®^Zθ (theta)) technology (Applied Biophysics Inc., Troy, NY, USA) followed by mathematical modeling as previously described [21,39]. Briefly, a 96-well array (96W20idf PET; Applied Biophysics Inc.) was first coated with 100 µM cysteine (50 µL/well; Applied Biophysics) for half an hour, followed by aspiration. Then, the array was coated with 0.02% gelatin (50 µL/well; Sigma, Burlington, MA, USA) for another half an hour, followed by aspiration. Next, human retinal endothelial cells (HRECs) obtained from Cell Systems (Kirkland, WA, USA) were seeded in Microvascular Endothelial Cell Growth Medium-2 BulletKit (Lonza, Walkersville, MD, USA; Catalog #: CC-3202 EGM-2 MV). After HRECs became confluent and formed a mature monolayer (as indicated by a capacitance below 20 nF), the culture media were replaced by media free of serum and growth factors for 10–12 h before applying different concentrations of CoCl_2_ (15862-1ML-F, Sigma, St. Louis, MO, USA). Thereafter, AC of 1 µA was subjected to HRECs cultured onto the electrode surfaces embedded in the bottom of each well to measure the overall Z with respect to time and frequency. Nine multifrequency measurements in a range from 250 Hz to 64,000 Hz were used. The Z value at each time point was normalized to the baseline Z acquired before the addition of CoCl_2_ and then plotted as a function of time.

Additionally, the ECIS system was used to dissect the overall Z into two parameters (resistance and capacitance) across the HREC monolayer. Following this, we further used the ECIS software to mathematical model transendothelial resistance across the HREC monolayer into three important components as previously described [40]. These components are R_b_ (an indicator for the integrity of the paracellular junctions between HRECs measured in Ω·cm^2^), alpha (α, an indicator of the basolateral attachment of HRECs to their extracellular matrix measured in Ω·cm^1/2^), and C_m_ (cell membrane’s capacitance which indicates changes in the HREC membrane morphology measured in µF/cm^2^). The data were collected either at the end or throughout the experiment by calculating the area under the curve (AUC).

### 5.2. Assessment of HREC Viability

The effect of different concentrations of CoCl_2_ on the viability of HRECs was assessed by lactate dehydrogenase (LDH) Cytotoxicity Assay (CyQUANT™; Invitrogen-C20300, Waltham, MA, USA). In this assay, HRECs were cultured in 96-well plates (1 × 10^4^/well), and after cells became confluent, the culture media were replaced by media free of serum and growth factors for 10–12 h before applying different concentrations of CoCl_2_ (0, 10, 100, and 1000 μM) for 24 h. After that, the amount of LDH released into the medium was determined per the manufacturer’s instructions.

### 5.3. Mitochondrial Bioenergetic Profiles

Seahorse bioanalyzer (XFe96, Agilent Technologies, Santa Clara, CA, USA) was used to measure the mitochondrial bioenergetic profile of HRECs by determining the oxygen consumption rate (OCR) as previously described [12,21]. HRECs (40,000 cells/well) were cultured in 96-XF tissue culture microplate (Agilent Technologies) using full media for 24 h. As HRECs reached confluency, the culture media were replaced by media free of serum and growth factors for 12–16 h, followed by CoCl_2_-treatment (0, 10, 100, and 1000 μM). At the end of the experiment, the culture media were replaced by XF assay media, then the Mito Stress test (Agilent Technologies) was performed. After the baseline was established, sequential injections of oligomycin (Olig, 1 µM), carbonyl cyanide-4 trifluoromethoxy phenylhydrazone (FCCP, 1 µM), and rotenone/Antimycin (1 µM each) were used to determine basal OCR, ATP-linked OCR, and maximal OCR.

### 5.4. Statistical Analysis

The two-tailed Student t-test or one-way analysis of variance (ANOVA) followed by the Tukey post-hoc test was used to determine differences between experimental groups. Graphical representations of *p* values are * *p* ≤ 0.05, ** *p* ≤ 0.01, *** *p* ≤ 0.001, and **** *p* ≤ 0.0001.

## Data Availability

Data associated with this manuscript are available upon request from corresponding author.
